# Conduction system pacing upgrade *versus* biventricular pacing on pacemaker-induced cardiomyopathy: a retrospective observational study

**DOI:** 10.3389/fphys.2024.1355696

**Published:** 2024-07-23

**Authors:** Ma Pei-pei, Chen Ying, Yang Yi-heng, Li Guo-cao, Ma Cheng-ming, Fa Qing, Gao Lian-jun, Xia Yun-long, Dong Ying-xue

**Affiliations:** Department of Cardiology, The First Affiliated Hospital of Dalian Medical University, Dalian, China

**Keywords:** pacemaker-induced cardiomyopathy, biventricular pacing, conduction system pacing, heart failure, cardiac resynchronization therapy

## Abstract

**Objective:** The feasibility of the conduction system pacing (CSP) upgrade as an alternative modality to the traditional biventricular pacing (BiVP) upgrade in patients with pacemaker-induced cardiomyopathy (PICM) remains uncertain. This study sought to compare two modalities of CSP (His bundle pacing (HBP) and left bundle branch pacing (LBBP)) with BiVP and no upgrades in patients with pacing-induced cardiomyopathy.

**Methods:** This retrospective analysis comprised consecutive patients who underwent either BiVP or CSP upgrade for PICM at the cardiac department from 2017 to 2021. Patients with a follow-up period exceeding 12 months were considered for the final analysis.

**Results:** The final group of patients who underwent upgrades included 48 individuals: 11 with BiVP upgrades, 24 with HBP upgrades, and 13 with LBBP upgrades. Compared to the baseline data, there were significant improvements in cardiac performance at the last follow-up. After the upgrade, the QRS duration (127.81 ± 31.89 vs 177.08 ± 34.35 ms, *p* < 0.001), NYHA class (2.28 ± 0.70 vs 3.04 ± 0.54, *p* < 0.05), left ventricular end-diastolic diameter (LVEDD) (54.08 ± 4.80 vs 57.50 ± 4.85 mm, *p* < 0.05), and left ventricular ejection fraction (LVEF) (44.46% ± 6.39% vs 33.15% ± 5.25%, *p* < 0.001) were improved. There was a noticeable improvement in LVEF in the CSP group (32.15% ± 3.22% vs 44.95% ± 3.99% (*p* < 0.001)) and the BiVP group (33.90% ± 3.09% vs 40.83% ± 2.99% (*p* < 0.001)). The changes in QRS duration were more evident in CSP than in BiVP (56.65 ± 11.71 vs 34.67 ± 13.32, *p* < 0.001). Similarly, the changes in LVEF (12.8 ± 3.66 vs 6.93 ± 3.04, *p* < 0.001) and LVEDD (5.80 ± 1.71 vs 3.16 ± 1.35, *p* < 0.001) were greater in CSP than in BiVP. The changes in LVEDD (*p* = 0.549) and LVEF (*p* = 0.570) were similar in the LBBP and HBP groups. The threshold in LBBP was also lower than that in HBP (1.01 ± 0.43 vs 1.33 ± 0.32 V, *p* = 0.019).

**Conclusion:** The improvement of clinical outcomes in CSP was more significant than in BiVP. CSP may be an alternative therapy to CRT for patients with PICM. LBBP would be a better choice than HBP due to its lower thresholds.

## Introduction

Long-term right ventricular pacing has been linked to cardiac dyssynchrony and pacemaker-induced cardiomyopathy (PICM) (Sharma et al., 2020). Upgrading to biventricular pacing (BiVP) was recommended for patients who developed PICM (Gierula et al., 2013). However, BiVP procedure failure and non-response were not uncommon (Khurshid et al., 2018).

Conduction system pacing (CSP), which includes His bundle pacing (HBP) and left bundle branch pacing (LBBP) modalities, has been shown to have a positive impact on cardiac performance (Herweg et al., 2021; Bednarek et al., 2023). The benefits of CSP upgrades in patients with PICM have also been reported (Yang et al., 2021). However, whether a CSP upgrade is a feasible alternative pacing modality to a BiVP upgrade still needs to be determined. This study aims to explore the different outcomes associated with varying upgrade modalities.

## Methods

### Population and study design

**FIGURE 1:**
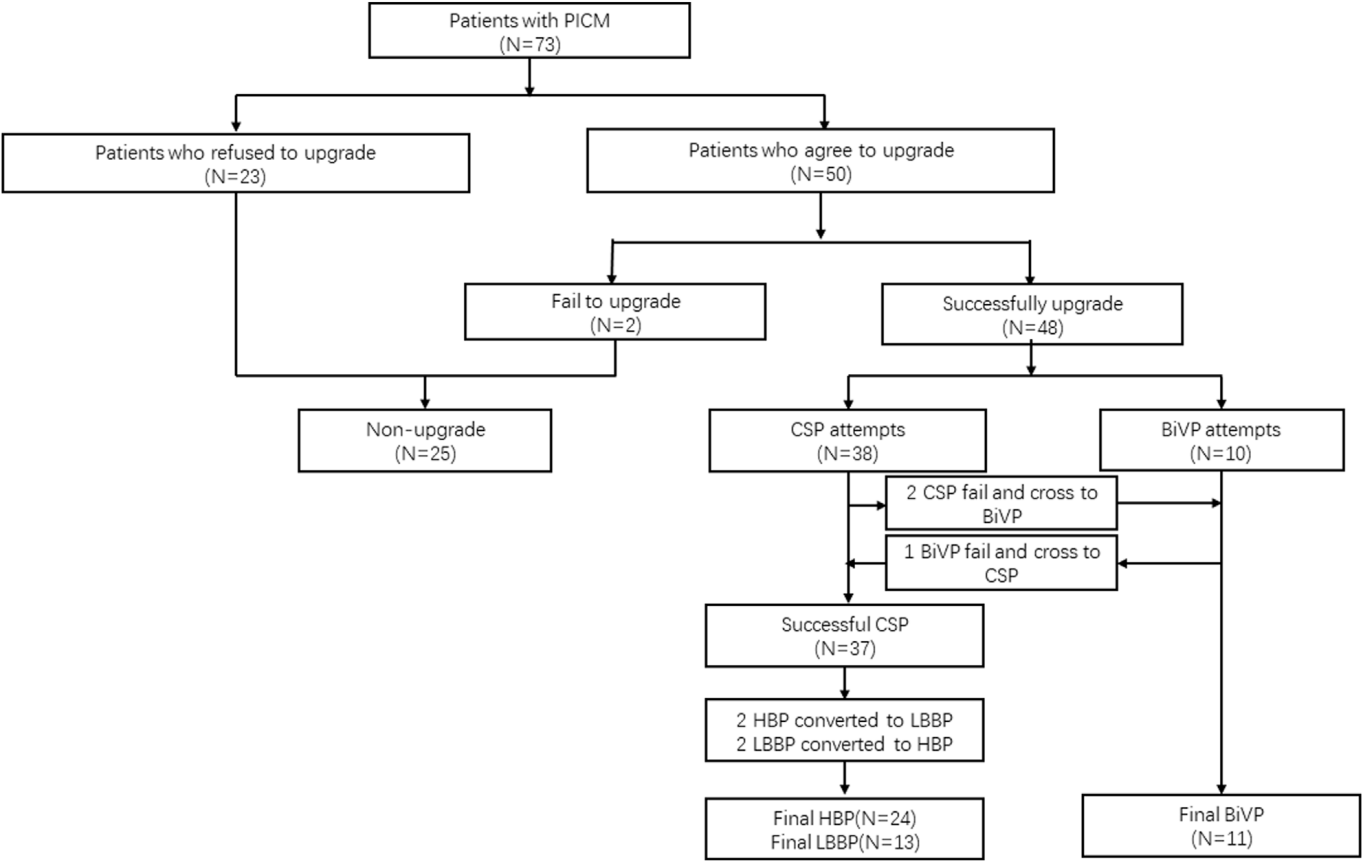
Flowchart of the study participants.

This study is a retrospective observational study. All patients with PICM were enrolled retrospectively from 2017 to 2021 at the First Affiliated Hospital of Dalian Medical University (FAHDMU). All patients were given a chance to choose an upgrade after being informed about the criteria for optimal pacing, success rate, factors for failure, complications, technical details for lead placement, and other pacing-related details. If the patient’s first choice failed, all patients agreed to switch to the alternative therapy (CSP to BiVP or *vice versa*). All patients provided consent for their respective treatments. The Ethics Committee of the FAHDMU approved the study and procedures (No. PJ-KS-KY-2023-365). The patient enrollment flowchart is shown in Figure 1.

### Measurements

Left ventricular end-diastolic diameter (LVEDD) and left atrial diameter (LAD) were measured based on the American Society of Echocardiography guidelines. LVEF was obtained using the biplane Simpson method. The vena contracta width with color flow Doppler was used to measure the maximum mitral regurgitation (MR) and tricuspid regurgitation (TR).

### Criteria and definitions

It is important to note that PICM definitions vary in different studies. In this study, we have established the following criteria for defining PICM: LVEF ≥50% before RVP implantation, the development of new-onset heart failure in patients with RVP >40% and LVEF ≤40%, and the absence of other causes of heart failure (Tops et al., 2009; Chen et al., 2013; Udo et al., 2015; Kiehl et al., 2016; Lu et al., 2022). Additionally, patients with AV node ablations were excluded. Left bundle branch area pacing capture was defined by an abrupt decrease in the Stim-LV active time (LVAT) of more than 10 ms and less than 75 ms, along with specific morphologies in Qr, qR, or rSRʹ in lead V1. HBP was not considered if a 1:1 His ventricular conduction was not noted during pacing at 110 beats per minute. LVEF greater than 50% and LVEDD less than 50 mm were considered complete LV reverse remodeling. The CSP response was defined as an absolute increase in LVEF by ≥ 5% after 1 year (Vijayaraman et al., 2018a; Abdin et al., 2022).

### Upgrading procedure

HBP and LBBP were performed using the select secure pacing lead (Model 3830, Medtronic Inc.) and a fixed-curve sheath (C315 HIS, Medtronic Inc.). His bundle electrograms were mapped in a unipolar configuration and recorded in the system (Prucka CardioLab, GE Healthcare) (Sundaram and Vijayaraman, 2020). The pacing rate was decreased to 30 bpm for an escape rhythm. Pacing mapping was conducted if no His bundle electrogram was detected. HBP was acceptable when the capture threshold was lower than 2.0 V/1.0 ms (Vijayaraman et al., 2018b). LBBP would be further performed if HBP was not detected. For patients with dependent ventricular pacing, right ventricular backup pacing was routinely performed if the threshold of HBP was higher than 2.0 v/0.5 ms. In patients with permanent AF who required a dual-chamber pacemaker, the 3830 lead was connected to the right atrial port, and the right ventricular lead remained in the RV port. In patients who received a new CRT defibrillator (D) or CRT pacemaker (P) device, the RA lead remained in the atrial port, and the RV lead remained in the RV port as a backup. The 3830 lead was connected to the left ventricular (LV) port.

The LV lead was implanted via the traditional coronary venous approach for BiVP implants. If possible, it is positioned using a standard technique in the lateral or posterolateral LV vein in patients with BiVP.

### Follow-up

All patients received optimal medical therapy for at least 3 months prior to the procedure. All patients with PICM were followed for at least 1 year, regardless of resynchronization upgrade. Clinical data were regularly collected for at least half a year. During the follow-up, a 12-lead electrocardiogram (ECG), echocardiography, postoperative complications, and pacemaker parameters were monitored.

### Statistical analysis

Statistical analyses were performed using SPSS 27.0. Continuous variables were expressed as the mean ± SD or median and were compared with an independent two-sample, paired *t*-test, or Wilcoxon test. Differences among groups were assessed using analysis of variance, or Kruskal–Wallis, depending on the presence of a normal distribution. Categorical variables were expressed as numbers (%) and were compared using Fisher’s exact test. *p* < 0.05 (two-tailed) was considered statistically significant.

## Results

### Baseline characteristics

During the study period, 73 patients were initially enrolled. Of these, 23 patients refused the upgrade, 2 patients tried to undergo the upgrade but failed, and 48 patients (48/50.96%) successfully underwent a resynchronization upgrade. Of these 48 upgraded patients, 24 received HBP, 13 received LBBP, and 11 received BiVP. During the procedure, two patients switched to LBBP due to a high threshold of over 2.0 V/0.4 ms, while another two patients reverted to HBP due to LBBP failure (Figure 1).

All PICM patients were followed up for 27.78 ± 9.69 months. There was no significant difference in gender, age, comorbidity, or ECG characteristics among those patients who underwent CSP, BiVP, and those without an upgrade (Table 1).

**TABLE 1:** Baseline characteristics of participants.

	Patients with CSP (n = 37)	Patients with BiVP (n = 11)	Patients without an upgrade (n = 25)	*p*-value
Male	17 (45.83)	4 (36.36)	11 (44.00)	0.850
Age	65.69 ± 14.85	59.86 ± 10.79	69.56 ± 15.83	0.360
Diabetes	10 (27.08)	2 (18.18)	10 (40.00)	0.346
Hypertension	24 (64.58)	5 (45.45)	17 (68.00)	0.412
Atrial fibrillation	15 (41.67)	3 (27.27)	11 (44.00)	0.621
Coronary heart disease	12 (31.25)	3 (27.27)	8 (32.00)	0.959
QRS duration (ms)	177.08 ± 34.35	181.71 ± 45.54	170.81 ± 31.29	0.375
LVEDD (mm)	57.50 ± 4.85	60.14 ± 6.18	55.67 ± 7.89	0.301
LAD (mm)	43.81 ± 7.39	43.57 ± 5.03	41.96 ± 7.02	0.501
LVEF (%)	33.15 ± 5.25	37.00 ± 3.96	35.15 ± 5.04	0.059
Mitral regurgitation	2.35 ± 0.43	2.28 ± 0.39	2.28 ± 0.39	0.853
Tricuspid regurgitation	1.53 ± 0.64	1.71 ± 0.49	1.70 ± 0.49	0.554
NYHA class	3.04 ± 0.54	3.27 ± 0.47	3.24 ± 0.60	0.234
NYHA II, n (%)	4 (10.81)	0 (0)	0 (0)	
NYHA III, n (%)	28 (75.68)	8 (72.73)	16 (64.00)	
NYHA IV, n (%)	5 (13.51)	3 (27.27)	9 (36.00)	
Follow-up year (year)	2.53 (1.54–3.30)	2.21 (1.55–3.11)	2.08 (1.69–3.05)	0.101
ACEI/ARB/ARNI (n, %)	32 (87.50)	10 (90.91)	21 (84.00)	0.900
β blockers (n, %)	33 (89.58)	10 (90.91)	19 (92 .00)	0.239
Diuretics (n, %)	33 (89.58)	11 (100)	23 (92.00)	0.858
Digoxin (n, %)	16 (43.85)	6 (54.55)	11 (44.00)	0.801

ACEI, angiotensin-converting enzyme inhibitor; ARB, angiotensin II receptor blocker; ARNI, angiotensin receptor-neprilysin inhibitor; LAD, left atrial diameter; LVEDD, left ventricular end-diastolic; LVEF, left ventricular ejection fraction.

### Procedural outcomes

Paced QRS duration was slightly shorter in HBP (102.08 ± 10.04 ms) and LBBP (113.95 ± 9.28 ms) than in BiVP (147.33 ± 12.67 ms). The pacing threshold was a little higher in HBP (1.33 ± 0.32V@0.4 ms) and BiVP (1.81 ± 0.60) than in LBBP (1.01 ± 0.43V@0.4 ms).

The pacing threshold remained stable during follow-up except for a significant increase in the pacing threshold (3.0V@1.0 ms) in one patient with HBP and BiVP. There was no infection, thrombosis, perforation, acute left heart failure, sudden death, or lead dislodgement after the upgrade. Procedural characteristics, including the complications, are shown in detail in Table 2.

**TABLE 2:** Characteristics of patients based on HBP, LBBP, and BiVP upgrades.

	HBP (n = 24)	LBBP (n = 13)	BiVP (n = 11)	*p*-value
Baseline QRS duration (ms)	179.21 ± 24.35	170.32 ± 13.34	182.00 ± 13.88	0.981
Paced QRS duration (ms)	102.08 ± 10.04	113.95 ± 9.28	147.33 ± 12.67	0.003
Threshold during procedure (V)	1.33 ± 0.32	1.01 ± 0.43	1.81 ± 0.60	0.702
Threshold after follow-up(V)	1.42 ± 0.52	0.90 ± 0.69	1.89 ± 0.63	0.464
Threshold increase ≥1 V	1 (2.70)	0	1 (4.00)	1.000
Ventricular pacing (%)	92.61 ± 2.98	93.81 ± 2.21	95.73 ± 2.31	0.661
Procedural complications	0	0	0	
Lead dislodgement	0	0	0	
Infection	0	0	0	
Perforation	0	0	0	

HBP, His bundle pacing; LBBP, left bundle branch pacing; BiVP, biventricular pacing.

### Clinical outcomes after follow-up

**FIGURE 2:**
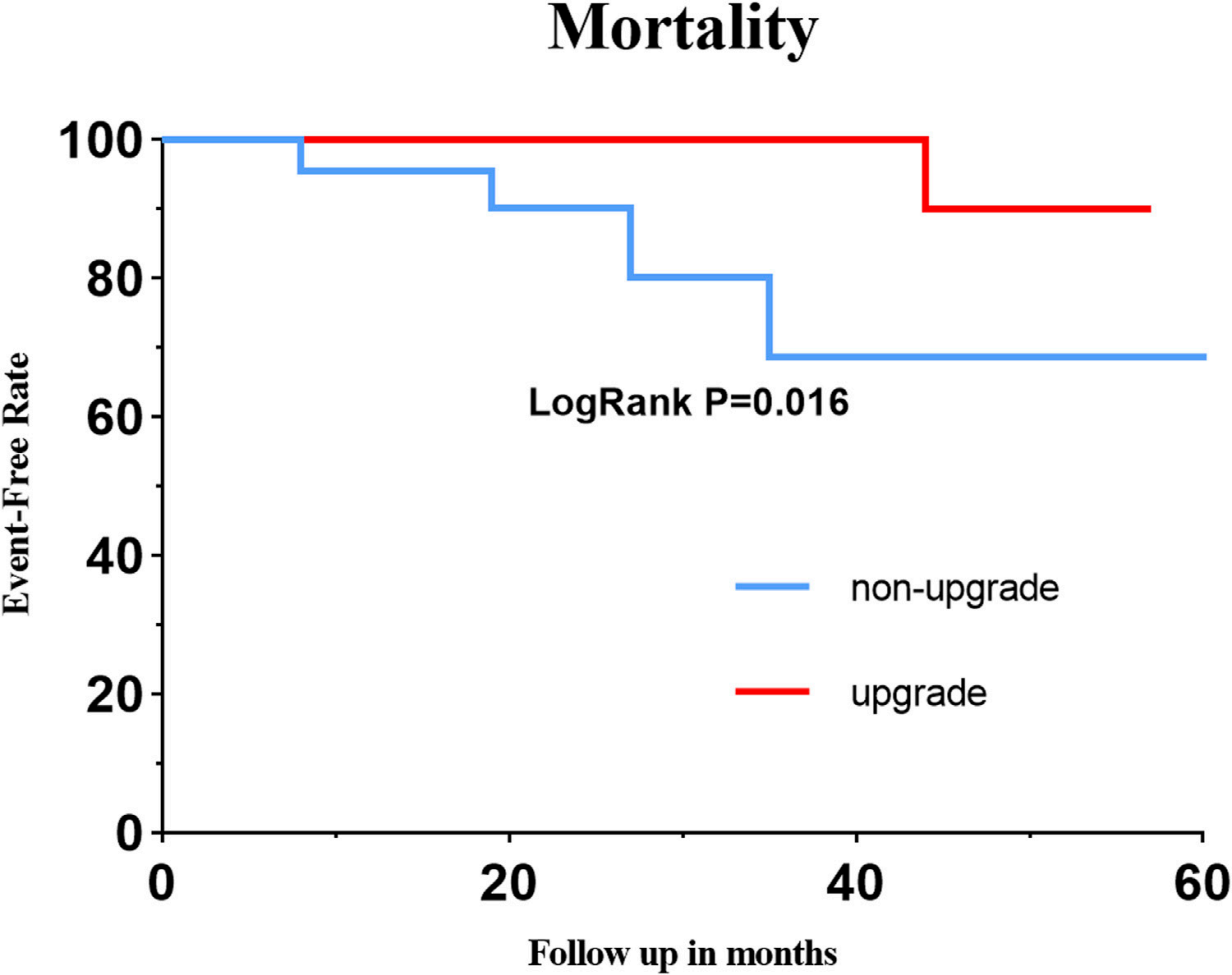
Kaplan–Meir curve of all-cause mortality.

Three patients (3/25, 12.00%) died of heart failure among those who did not undergo an upgrade, and no cardiac death occurred in patients whose received the upgrade. Four patients with CSP were re-hospitalized, and one patient died of kidney failure 2 years after the upgrade. The Kaplan–Meir curve of all-cause mortality is shown in Figure 2.

**FIGURE 3:**
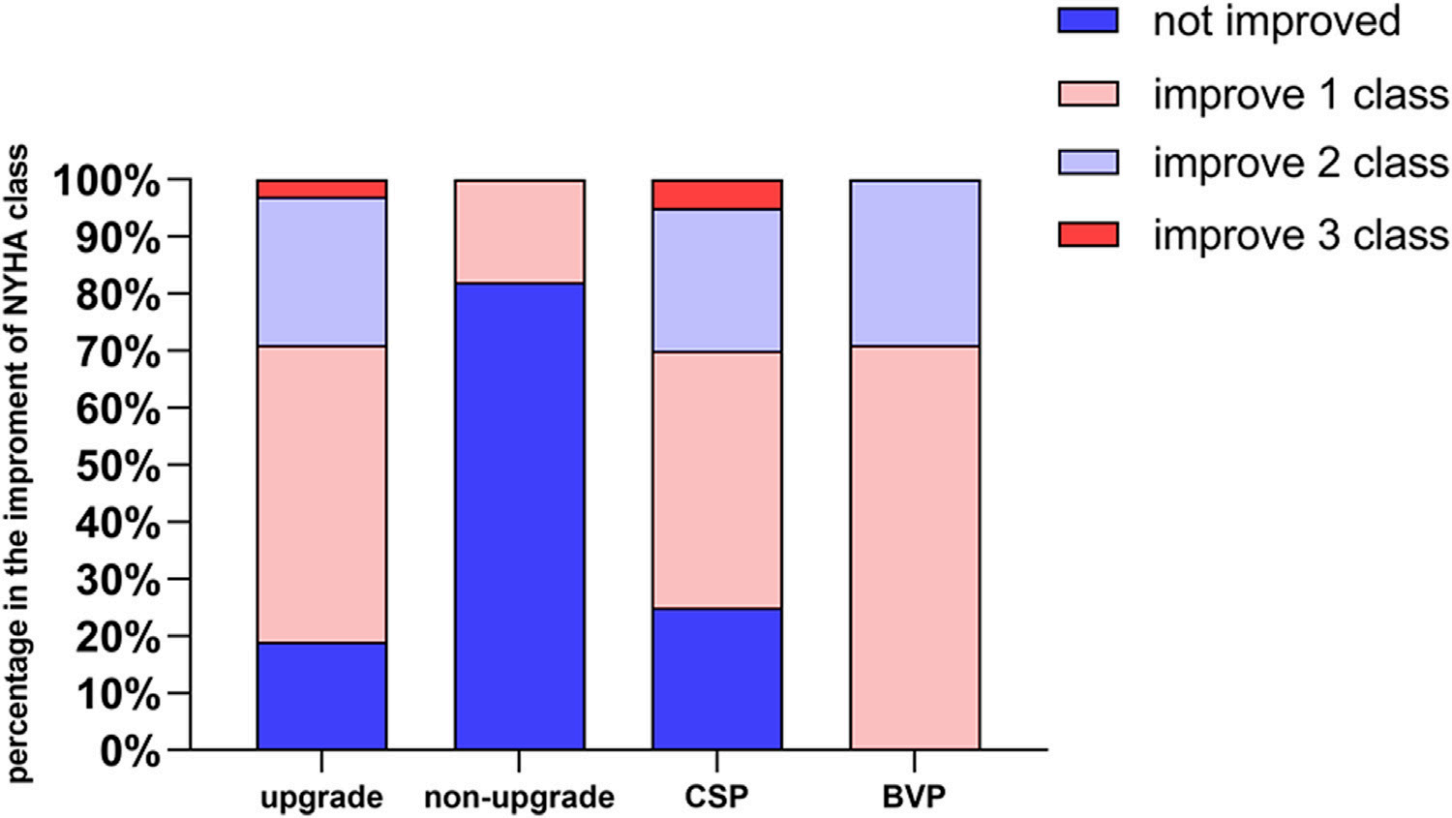
NYHA class change after follow-up.

The response in CSP was similar to that in BiVP (95.83% vs 81.82%, *p* = 0.154). Complete remodeling was more common in CSP than in BiVP patients (66.67% vs 27.27%, *p* = 0.040). The median time to complete reverse remodeling was 6.21 ± 3.57 months. NYHA class improved ≥1 class in CSP was similar to that in BiVP (*p* = 0.368), as shown in Figure 3. NYHA class improvement was not found in four patients with NYHA II and three with NYHA III. However, LVEF improvement was noticeable in seven patients (31.00% ± 3.37% vs 45.75% ± 9.74%, *p* = 0.037).

### Echocardiographic outcomes after follow-up

It is fascinating to note that patients who experienced a decrease in the ejection fraction at baseline and received upgrades demonstrated significant improvement compared to the non-upgraded group (33.15% ± 5.25% vs 44.46% ± 6.39%, *p* < 0.001). Furthermore, there was a noticeable improvement in LVEF in the CSP group (32.15% ± 3.22% vs 44.95% ± 3.99%, *p* < 0.001) and the BiVP group (33.90% ± 3.09% vs 40.83% ± 2.99%, *p* < 0.001). The upgrade group also experienced a decrease in LVEDD (57.50 ± 4.85 vs 54.08 ± 4.80 mm, *p* < 0.05). Notably, upgraded patients generally showed improvement in tricuspid regurgitation (1.15 ± 0.46 vs 1.57 ± 0.60, *p* = 0.018). Additionally, significant improvements in QRS duration and NYHA class were observed in the upgraded group during follow-up compared to the baseline.

**FIGURE 4:**
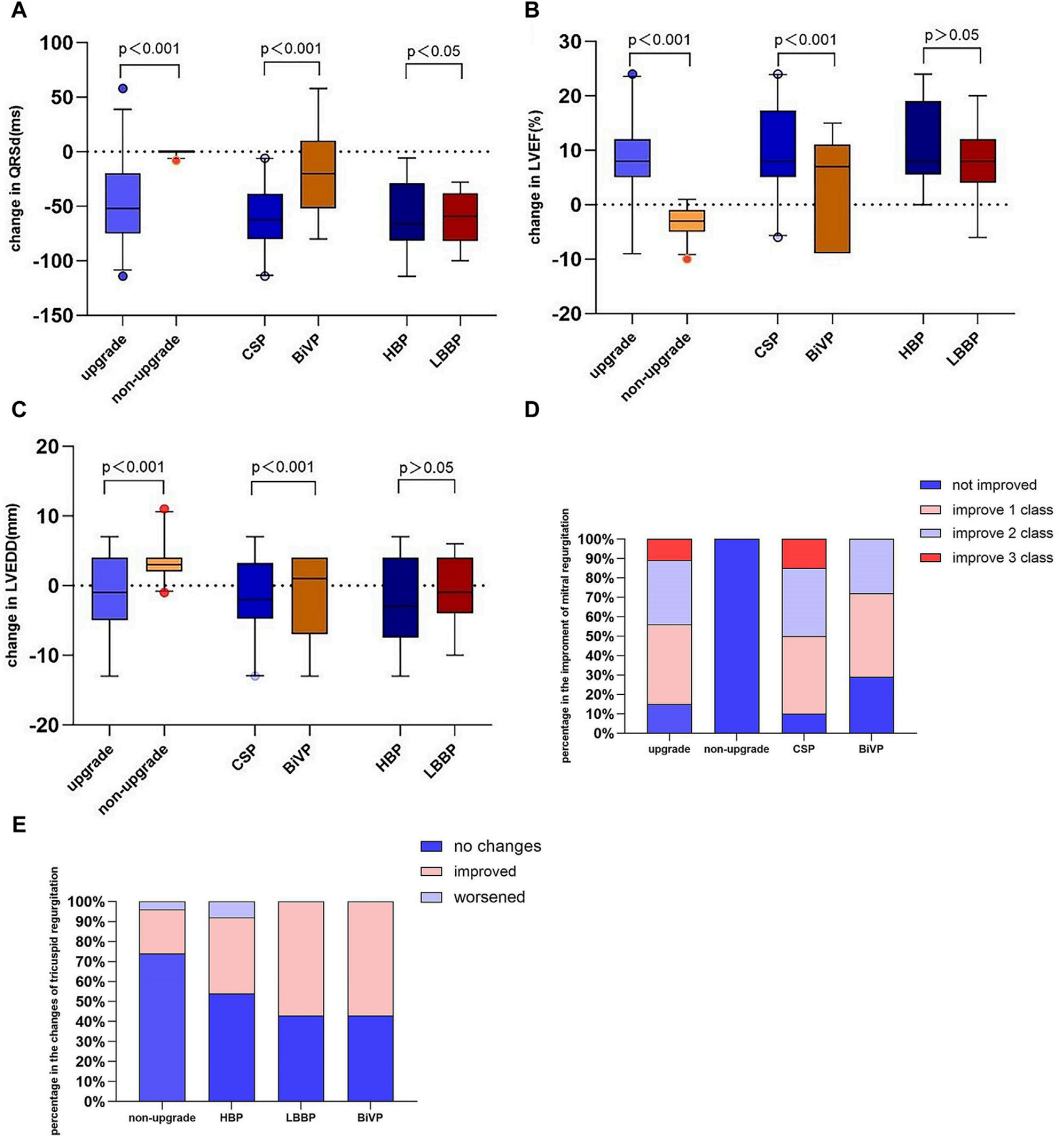
Change in QRS duration **(A)**, LVEF **(B)**, LVEDD **(C)**, mitral regurgitation **(D),** and tricuspid regurgitation **(E)**. LVEF, left ventricular ejection fraction; LVEDD, left ventricular end-diastolic.

The changes in QRS duration were more evident in CSP than in BiVP (56.65 ± 11.71 vs 34.67 ± 13.32, *p* < 0.001) (Figure 4A). Similarly, the changes in LVEF (12.8 ± 3.66 vs 6.93 ± 3.04, *p* < 0.001) and LVEDD (5.80 ± 1.71 vs 3.16 ± 1.35, *p* < 0.001) were greater in CSP than in BiVP (Figures 4B,C). There was no statistically significant change in LVEF (11.62% ± 3.73% vs 10.55% ± 3.01%, *p* = 0.570) and LVEDD (6.19 ± 8.34 vs 8.70 ± 6.45 mm, *p* = 0.549) between HBP and LBBP. Regurgitation deterioration was found in one patient after HBP.

## Discussion

In this single-center, retrospective study, we discovered that the echocardiographic response and complete reverse remodeling of the left ventricle were significantly higher in patients who underwent CRT with a CSP upgrade than those with a BiVP upgrade. Additionally, we observed similar clinical improvements between patients who received an upgrade to HBP and those who underwent an LBBP upgrade in individuals with PICM.

### Different options for cardiac resynchronization in patients with PICM

Previous studies have shown that the BiVP upgrade had positive outcomes for patients with PICM. Witte et al. (2006) found that the BiVP upgrade helped reverse left ventricular dilatation and dysfunction and reduce mitral regurgitation. In a prospective cohort study, improvements in left ventricular ejection fraction (LVEF) (33.3% ± 5.2% to 47.6% ± 9.3%, *p* < 0.001) and NYHA grade were observed after the BiVP upgrade (Schwerg et al., 2015). Consistent with these findings, our present study also demonstrated significantly improved LVEF (33.90% ± 3.09% vs 40.83% ± 2.99%, *p* < 0.001) and NYHA class. The longer-paced QRS duration after RVP and the more obvious QRS duration shortening after the BiVP upgrade might be associated with a more favorable CRT response (Hsing et al., 2011). Nevertheless, a substantial portion of individuals undergoing BiVP might not attain any clinical or echocardiographic advantages, and a few might even deteriorate (European Heart Rhythm et al., 2012).

Previous research had demonstrated notable enhancements in cardiac performance following CSP. CSP was effective in electrical resynchronization, narrowed QRS complex, and improved LVEF in patients with PICM (Vijayaraman et al., 2019; Gardas et al., 2022). However, there was a lack of direct comparative studies between BiVP and CSP upgrades. In the present study, we demonstrated a much more significant reduction in QRS duration with CSP compared to BiVP. It was observed for the first time that the improvements in LVEF (ΔLVEF) and LVEDD (ΔLVEDD) were significantly higher in CSP than in BiVP, and the improvements in LVEF and LVEDD were similar between LBBP and HBP.

The present study also demonstrated a similar risk in tricuspid regurgitation with LBBP and BiVP, which was consistent with the findings of Li et al. (2022).

When addressing heart failure caused by the pacing modality, physiological pacing was the preferred option for restoring normal cardiac function in patients with PICM. Clinical trials had shown the remarkable effectiveness of physiological pacing in improving cardiac function over both short- and long-term periods when compared to traditional right ventricular pacing (RVP) (Kronborg et al., 2014; Huang et al., 2017). A recent study indicated that the incidence of PICM in HBP was significantly lower than that in RVP (2% vs 22%, *p* = 0.04) (Vijayaraman et al., 2018c), suggesting that HBP might be the first-line recommendation for patients at high risk of PICM.

### Feasibility and safety of an upgrade in patients with PICM

BiVP procedures account for nearly a quarter of all CRT procedures. While BiVP might reduce the risk of death or heart failure by 33%, the upgrade procedure might also be associated with more complications (Valls Bertault et al., 2004; Poole et al., 2010; Linde et al., 2018). Cheung et al. (2017) examined the outcomes of CRT upgrade procedures compared to *de novo* CRT procedures. The findings revealed that upgrade procedures were linked to higher mortality rates, increased incidents of cardiac perforation, and a greater need for lead revision (Cheung et al., 2017). However, a meta-analysis discovered that the average complication rates for patients undergoing biventricular upgrade were 2% for pneumothorax, 1.4% for tamponade, and 3.7% for infection during a mean follow-up period of 24 months (Kaza et al., 2023). In addition, the meta-analysis reported that 3.3% of patients with biventricular upgrades and 1.8% of patients with CSP upgrades experienced complications related to lead placement. A two-center, observational, retrospective study found that the pacing threshold was 1.62 ± 1.0 V after 5 years of follow-up in HBP (Vijayaraman et al., 2018c). Guo et al. (2020) showed that the LBBP had satisfactory and stable lead parameters at 12 months. Similarly, our study showed a stable pacing threshold in patients with CSP after nearly 2 years of follow-up.

Typically, lower and more consistent pacing thresholds and higher R-wave amplitudes were observed with LBBP instead of HBP (Chen et al., 2019). This indicates that LBBP may offer a promising alternative for providing physiological pacing with improved stability in pacing thresholds (Wu et al., 2019). Our study also revealed a lower pacing threshold (*p* = 0.018) in LBBP than in HBP. To comprehensively assess the safety and feasibility of CSP and BiVP upgrades, randomized clinical trials with long-term follow-up would be essential.

Padala et al. (2020) reported a successful delivery of LBBP in 89% of patients. Ye et al. (2021) showed that the LBBP upgrade succeeded in 95% of patients with PICM. Our study also demonstrated a similar success ratio of the CSP upgrade (95.83%). The high success rate was attributed to the adoption of both HBP and LBBP in this study, as well as the operators’ experience.

### Clinical performance of CSP in patients with PICM

In the treatment of patients with PICM, CSP plays a vital role. Huang et al. revealed that in patients with clinically symptomatic heart failure and LVEF <50%, HBP upgrade was feasible in 88.9% of cases, leading to significant improvement in left ventricular function and remodeling (Shan et al., 2018). For patients with infra-nodal atrioventricular block, LBBP emerged as a feasible option for pacing beyond the block site, serving as a reasonable alternative to cardiac resynchronization pacing via a coronary sinus lead. Furthermore, Qian et al. (2021) showed that 69.2% of LBBP patients with heart failure after right ventricular pacing experienced an absolute increase in LVEF of over 5%.

Gardas et al. (2022) found that the LVEF response was higher in HBP than in BiVP (92.3% vs 81.2%, *p* < 0.05). In our study, we observed that the echo response in CSP was similar to BiVP (95.83% vs 81.82%, *p* = 0.154). However, complete left ventricular reverse remodeling was more common in CSP than in BiVP (66.67% vs 27.27%, *p* = 0.040). The lack of complete cardiac reverse remodeling in PICM patients can be attributed to the existence of multiple myocardial lesions and an increase in permanent myocardial scar formation. Guan et al. (2022) demonstrated that a history of heart failure was associated with outcomes following CSP. These findings strongly suggest that CSP upgrades should be performed before irreversible heart failure develops.

## Limitations

This study was an observational, single-center retrospective study. BiVP or CSP was chosen in this observational study based on patients’ preferences. Therefore, the upgrade strategy was not randomized, and the results should be interpreted with caution. Prospective, randomized trials with an extended follow-up period are crucial to compare BiVP and CSP and validate the observed outcomes identified in this study.

## Conclusion

Both CSP and BiVP upgrades improved cardiac performance in PICM. The improvement in cardiac performance was more significant in CSP than in BiVP. HBP and LBBP had similar improvements in left ventricular performance, while LBBP would be an optimal choice for a lower pacing threshold compared with HBP.

## Data Availability

The raw data supporting the conclusion of this article will be made available by the authors, without undue reservation.
