# Supplementary material for: Conduction system pacing upgrade versus biventricular pacing on pacemaker-induced cardiomyopathy: a retrospective observational study
Source: Front Physiol. 2024 Jul 23;15:1355696. doi: 10.3389/fphys.2024.1355696 (PMC11300236; doi:10.3389/fphys.2024.1355696)
Supplement: Supplementary file 1 [file DataSheet1.docx]

**Table S1. Demographics of non-responders.**

| Patient | Group | Age | Gender | Paced QRS | | LVEF | | NYHA class | |
| --- | --- | --- | --- | --- | --- | --- | --- | --- | --- |
|  |  |  |  | Pre-upgrade | Post-upgrade | Pre-upgrade | Post-upgrade | Pre-upgrade | Post-upgrade |
| 1 | CSP | 82 | Male | 152 | 114 | 36 | 30 | 4 | 2.5 |
| 2 | CSP | 70 | Female | 166 | 110 | 32 | 32 | 3 | 3 |
| 3 | BiVP | 66 | Male | 182 | 160 | 40 | 31 | 3 | 1.5 |
| 4 | BiVP | 66 | Male | 180 | 163 | 39 | 30 | 3 | 1.5 |

LVEF: left ventricular ejection fraction, NYHA: [New York Heart Association Classification](https://manual.jointcommission.org/releases/TJC2018A/DataElem0439.html)

**Table S2. Cardiac function and echocardiographic parameters** **in baseline and during follow-up**

|  | baseline | during follow-up | *P* value |
| --- | --- | --- | --- |
| QRS duration in upgrade | 177.08±34.35 | 127.81±31.89 | ＜0.001 |
| QRS duration in non-upgrade | 170.81±31.29 | 167.33±38.67 | ＞0.05 |
| QRS duration in CSP | 175.60±12.0 | 115.95±11.40 | ＜0.001 |
| QRS duration in BiVP | 182.00±13.88 | 147.33±12.67 | ＜0.001 |
| NYHA class in upgrade | 3.04±0.54 | 2.28±0.70 | ＜0.001 |
| NYHA class in non-upgrade | 3.24±0.60 | 3.44±0.76 | ＞0.05 |
| LVEF in upgrade | 33.15±5.25 | 44.46±6.39 | ＜0.001 |
| LVEF in non-upgrade | 35.15±5.04 | 30.57±7.65 | ＞0.05 |
| LVEF in CSP | 32.15±3.22 | 44.95±3.99 | ＜0.001 |
| LVEF in BiVP | 33.90±3.09 | 40.83±2.99 | ＜0.001 |
| LVEDD in upgrade | 57.50±4.85 | 54.08±4.80 | ＜0.05 |
| LVEDD in non-upgrade | 55.67±7.89 | 59.87±6.25 | ＞0.05 |
| LVEDD in CSP | 59.80±1.93 | 52.00±3.35 | ＜0.001 |
| LVEDD in BiVP | 60.83±1.46 | 56.67±3.20 | ＞0.05 |
| TR in upgrade | 1.15±0.46 | 1.57±0.60 | ＜0.05 |

LVEF: left ventricular ejection fraction; LVEDD, left ventricular end-diastolic diameter; TR: tricuspid regurgitation.
